# Supplementary material for: A Likelihood Approach for Real-Time Calibration of Stochastic Compartmental Epidemic Models
Source: PLoS Comput Biol. 2017 Jan 17;13(1):e1005257. doi: 10.1371/journal.pcbi.1005257 (PMC5240920; doi:10.1371/journal.pcbi.1005257)
Supplement: S1 File — (TAR.GZ) [file pcbi.1005257.s014.tar.gz › HSPH_Online-SI-Revision/IPoi09/n10-severe/n10-severe_BENCH_table-4.pdf]

1	R0	{1.68952, 11.8402}	{2.18903, 15.1095}	{{2.04669, 13.1693}, {2.24242, 15.7579}}	{29.5655, 27.6122}	inf	719.	1472.93	{797., 1914.}	111.63	0.323655	newly	473.	716.443	{458., 888.}	60.2502	0.21429	Reff	1.34131	1.33153	{0.849346, 1.83158}	23.1261	ar	6872.	8412.18	{8219., 8642.}	22.4124
2	R0	{1.71739, 9.62661}	{1.89283, 10.4879}	{{1.70777, 8.49961}, {2.15363, 13.5067}}	{10.3609, 16.9805}	inf	504.	950.966	{292., 1578.}	105.411	0.328911	newly	426.	656.915	{250., 934.}	69.6984	0.267433	Reff	1.47352	1.39907	{0.97751, 1.8543}	18.1482	ar	6874.	7654.19	{7003., 8458.}	11.7493
3	R0	{1.48936, 6.23975}	{1.74198, 7.14182}	{{1.7356, 7.10024}, {1.739, 7.10524}}	{16.9618, 14.4568}	inf	371.	822.426	{431., 1141.}	130.063	0.366335	newly	491.	856.021	{533., 1081.}	83.1564	0.279722	Reff	1.29008	1.24361	{0.902859, 1.5411}	16.3571	ar	5666.	7104.43	{6928., 7224.}	25.387
4	R0	{1.62394, 7.51135}	{2.2611, 18.8812}	{{2.25962, 18.788}, {2.26161, 19.3849}}	{39.2353, 151.369}	inf	401.	1124.63	{309., 1919.}	194.023	0.462	newly	435.	454.831	{137., 730.}	43.5145	0.246845	Reff	1.42192	1.6992	{1.17681, 2.14446}	26.9903	ar	6417.	8555.12	{8463., 8652.}	33.3196
5	R0	{1.53632, 8.57652}	{2.31153, 18.7591}	{{2.21213, 17.5123}, {2.39393, 19.7817}}	{50.4586, 118.727}	inf	485.	1430.82	{636., 2117.}	200.647	0.454061	newly	460.	566.785	{286., 779.}	43.301	0.187662	Reff	1.29543	1.54821	{1.08038, 2.07945}	29.8613	ar	6260.	8647.45	{8382., 8824.}	38.1381
6	R0	{1.47166, 5.7434}	{2.26986, 18.4991}	{{2.18067, 16.87}, {2.37462, 19.3966}}	{54.2382, 222.094}	inf	232.	803.625	{255., 1675.}	251.926	0.482071	newly	328.	346.766	{110., 632.}	44.8599	0.215844	Reff	1.32906	1.88803	{1.53834, 2.16681}	42.5532	ar	5650.	8542.22	{8373., 8724.}	51.1897
7	R0	{1.48179, 8.87369}	{2.14233, 14.4636}	{{2.04669, 13.0262}, {2.22867, 15.6411}}	{44.5775, 62.9945}	inf	510.	1470.84	{1062., 1804.}	191.55	0.459378	newly	397.	735.578	{598., 883.}	89.3931	0.280167	Reff	1.14379	1.24665	{0.809057, 1.65969}	26.4063	ar	5233.	8296.53	{8091., 8510.}	58.5425
8	R0	{1.53669, 6.47807}	{2.60499, 16.8175}	{{2.29055, 13.7413}, {2.76849, 18.33}}	{69.5198, 159.607}	inf	392.	1340.08	{496., 2152.}	246.654	0.502255	newly	477.	648.078	{254., 950.}	55.8932	0.217141	Reff	1.32417	1.9613	{1.55213, 2.43214}	48.2473	ar	5934.	9038.62	{8644., 9276.}	52.3192
9	R0	{1.57227, 6.20989}	{1.47895, 4.48038}	{{1.42949, 4.00571}, {1.50982, 4.79544}}	{6.3959, 28.608}	inf	215.	325.467	{113., 569.}	78.9767	0.263448	newly	311.	599.733	{229., 992.}	105.188	0.294014	Reff	1.49381	1.28863	{1.086, 1.44188}	13.7358	ar	6136.	5582.47	{4895., 5990.}	9.02108
10	R0	{1.54936, 6.50938}	{1.97926, 6.94846}	{{1.81768, 5.68021}, {2.28593, 9.04943}}	{27.7469, 17.4043}	inf	423.	854.847	{172., 1331.}	125.011	0.372933	newly	555.	1002.12	{263., 1518.}	101.945	0.328824	Reff	1.36483	1.5584	{1.07519, 2.02015}	24.469	ar	6275.	7868.39	{7380., 8650.}	25.3927
11	R0	{1.42179, 3.18601}	{1.50821, 4.77992}	{{1.50982, 4.79544}, {1.50982, 4.79544}}	{6.07849, 50.0285}	inf	124.	453.	{183., 692.}	265.819	0.520275	newly	375.	744.462	{371., 1014.}	102.154	0.28782	Reff	1.33549	1.23358	{1.05159, 1.41274}	10.2828	ar	5322.	5842.96	{5611., 6044.}	9.78883
12	R0	{1.48035, 6.46183}	{1.51547, 4.864}	{{1.50982, 4.79544}, {1.50982, 4.79544}}	{2.64727, 25.3199}	inf	191.	463.69	{183., 695.}	146.489	0.362347	newly	266.	745.862	{371., 1014.}	181.877	0.427041	Reff	1.39049	1.24246	{1.04948, 1.42618}	12.0661	ar	5793.	5932.72	{5611., 6259.}	4.14531
13	R0	{1.75755, 8.53854}	{2.44584, 17.1759}	{{2.315, 15.4658}, {2.64373, 19.7855}}	{39.1618, 101.157}	inf	456.	1034.02	{228., 1969.}	150.909	0.426555	newly	444.	474.774	{137., 815.}	52.2886	0.312892	Reff	1.57195	1.97038	{1.47327, 2.47638}	29.8957	ar	6832.	8863.74	{8578., 9137.}	29.7386
14	R0	{1.47355, 4.89451}	{2.82644, 16.3966}	{{2.688, 14.8279}, {2.96398, 17.7348}}	{91.8117, 235.}	inf	339.	1114.8	{417., 2027.}	234.409	0.472512	newly	540.	592.775	{238., 1003.}	44.0402	0.202132	Reff	1.31367	2.29209	{1.86115, 2.68182}	74.4797	ar	5710.	9266.39	{9138., 9387.}	62.2836
15	R0	{1.62071, 5.98665}	{1.44232, 2.62665}	{{1.43965, 2.60601}, {1.43965, 2.60601}}	{11.0066, 56.1249}	inf	269.	426.6	{135., 577.}	78.7212	0.280406	newly	451.	1165.68	{542., 1475.}	164.922	0.412314	Reff	1.51245	1.1242	{0.947722, 1.3658}	25.6702	ar	6635.	5470.76	{5254., 5744.}	17.5469
16	R0	{1.67268, 7.7256}	{1.74613, 5.92349}	{{1.74092, 5.83416}, {1.75359, 6.01426}}	{4.39138, 23.3265}	inf	657.	855.319	{551., 1040.}	35.9953	0.132068	newly	678.	917.766	{582., 1219.}	41.891	0.148478	Reff	1.34935	0.845593	{0.645533, 1.08337}	38.1509	ar	6947.	7119.87	{6977., 7294.}	2.66486
17	R0	{1.67805, 11.1417}	{1.48533, 4.4789}	{{1.44762, 4.00571}, {1.50982, 4.79544}}	{11.5532, 59.8005}	inf	455.	473.265	{270., 640.}	26.4706	0.116999	newly	326.	700.294	{415., 956.}	115.68	0.316227	Reff	1.48021	0.941087	{0.750591, 1.24293}	36.4219	ar	6625.	5676.59	{5261., 5990.}	14.3156
18	R0	{1.66743, 9.99046}	{1.80262, 8.63749}	{{1.67405, 7.28757}, {1.93816, 10.0503}}	{8.10747, 13.9728}	inf	487.	836.795	{370., 1364.}	83.9509	0.254564	newly	415.	712.977	{365., 966.}	78.6802	0.244013	Reff	1.42932	1.3567	{1.00675, 1.70345}	15.6221	ar	6899.	7379.2	{6719., 7834.}	8.3039
19	R0	{1.70787, 9.85478}	{2.41579, 19.3529}	{{2.42205, 19.4475}, {2.42205, 19.4475}}	{41.4502, 96.381}	inf	650.	1326.45	{329., 2198.}	125.454	0.401428	newly	499.	521.774	{146., 806.}	42.7177	0.26718	Reff	1.37449	1.7244	{1.20061, 2.28286}	33.1061	ar	6867.	8794.32	{8709., 8880.}	28.0664
20	R0	{1.53809, 10.5848}	{1.85317, 13.0308}	{{1.78373, 11.799}, {1.94617, 14.0718}}	{20.485, 23.832}	inf	420.	930.263	{354., 1321.}	131.358	0.356307	newly	300.	525.211	{231., 699.}	86.0877	0.278858	Reff	1.34152	1.33484	{1.00177, 1.69294}	17.9386	ar	6108.	7513.47	{7144., 8544.}	23.0104
21	R0	{1.59925, 6.74479}	{1.58235, 4.27715}	{{1.53501, 4.02511}, {1.61691, 4.41442}}	{3.04025, 36.5858}	inf	387.	559.6	{226., 878.}	68.418	0.323665	newly	488.	970.289	{533., 1341.}	109.713	0.397118	Reff	1.42989	1.21304	{0.943878, 1.49791}	17.054	ar	6411.	6153.	{5800., 6728.}	7.23063
22	R0	{1.38779, 4.40977}	{1.36173, 3.24525}	{{1.36035, 3.22945}, {1.36035, 3.22945}}	{1.9978, 26.4076}	inf	183.	284.842	{171., 402.}	58.8151	0.190241	newly	337.	551.579	{309., 881.}	67.1716	0.209744	Reff	1.26761	0.912819	{0.743295, 1.17235}	27.9888	ar	5154.	4895.16	{4643., 5238.}	5.76354
23	R0	{1.53657, 7.50352}	{1.74324, 7.15833}	{{1.7356, 7.10024}, {1.739, 7.10524}}	{13.4503, 5.68004}	inf	441.	890.925	{568., 1095.}	107.723	0.32074	newly	429.	876.66	{642., 1058.}	107.886	0.31665	Reff	1.31868	1.07577	{0.761755, 1.46024}	22.9978	ar	5928.	7093.89	{6908., 7246.}	19.6675
24	R0	{1.71131, 6.24526}	{2.49762, 15.7477}	{{2.0072, 10.6431}, {2.76849, 18.33}}	{45.9916, 152.155}	inf	217.	586.708	{112., 1113.}	191.212	0.453374	newly	358.	333.556	{65., 597.}	50.8225	0.341439	Reff	1.60743	2.25709	{1.78597, 2.63144}	40.8382	ar	6924.	8868.6	{8023., 9296.}	28.0849
25	R0	{1.4412, 3.07729}	{1.32313, 3.54039}	{{1.31535, 3.47086}, {1.31535, 3.47086}}	{8.38475, 15.049}	inf	56.	112.8	{9., 232.}	142.857	0.539433	newly	207.	271.7	{18., 556.}	84.058	0.51222	Reff	1.41454	1.26404	{1.18855, 1.30864}	11.026	ar	5810.	4401.2	{4022., 5190.}	24.2478
26	R0	{1.4159, 4.93858}	{1.80308, 5.98474}	{{1.74592, 5.58477}, {1.80312, 5.98216}}	{27.3449, 21.1834}	inf	225.	622.593	{93., 1000.}	196.346	0.469789	newly	397.	866.833	{178., 1323.}	136.165	0.385388	Reff	1.30079	1.50617	{1.22215, 1.76706}	19.7701	ar	5255.	7340.43	{7007., 7533.}	39.6846
27	R0	{1.57465, 7.05414}	{1.4594, 3.59346}	{{1.43579, 3.38828}, {1.47608, 3.83841}}	{8.62478, 49.0589}	inf	216.	325.	{120., 614.}	75.1462	0.265906	newly	292.	755.895	{385., 1102.}	164.672	0.401492	Reff	1.4934	1.26629	{1.08531, 1.40952}	15.2543	ar	6359.	5497.63	{5125., 5757.}	14.9875
28	R0	{1.41422, 4.62402}	{2.48267, 12.5248}	{{2.16209, 9.98575}, {2.84915, 15.0981}}	{75.5507, 170.863}	inf	138.	480.875	{182., 889.}	252.174	0.479105	newly	285.	370.563	{150., 669.}	58.5855	0.221873	Reff	1.35298	2.29225	{1.93128, 2.68703}	69.4215	ar	5485.	8890.55	{8345., 9293.}	62.0884
29	R0	{1.41901, 3.63017}	{2.39258, 8.4644}	{{1.90341, 5.70177}, {2.74056, 10.6536}}	{68.609, 133.168}	inf	127.	453.226	{54., 969.}	279.959	0.57668	newly	382.	570.597	{79., 1079.}	85.9441	0.36252	Reff	1.3631	2.23395	{1.78369, 2.59637}	64.1364	ar	5979.	8543.77	{7475., 9233.}	46.1114
30	R0	{1.60719, 4.72984}	{2.57086, 10.5096}	{{2.19024, 8.18891}, {2.93502, 12.6864}}	{59.9599, 122.199}	inf	120.	289.426	{40., 754.}	170.723	0.394506	newly	274.	293.632	{57., 687.}	68.1355	0.331874	Reff	1.56171	2.47754	{2.076, 2.79531}	58.6431	ar	6479.	8960.1	{8476., 9381.}	38.2945
31	R0	{1.38175, 6.26468}	{1.43579, 3.38828}	{{1.43579, 3.38828}, {1.43579, 3.38828}}	{3.91098, 45.9146}	inf	357.	414.957	{290., 527.}	29.1925	0.160092	newly	432.	782.913	{552., 1012.}	90.0463	0.301396	Reff	1.2035	0.948427	{0.779203, 1.18754}	23.0397	ar	5397.	5410.48	{5138., 5724.}	2.90983
32	R0	{1.4947, 8.8847}	{1.81483, 10.4627}	{{1.80156, 10.0119}, {1.81647, 10.5184}}	{21.4177, 17.7607}	inf	462.	1061.64	{719., 1262.}	130.62	0.354174	newly	361.	687.267	{499., 810.}	91.1665	0.273646	Reff	1.18634	0.953343	{0.726043, 1.26553}	24.9728	ar	5407.	7377.51	{7222., 7520.}	36.4437
33	R0	{1.55891, 8.28978}	{2.40039, 19.1999}	{{2.24732, 17.1868}, {2.42625, 19.5287}}	{53.9789, 131.609}	inf	446.	1471.85	{194., 2270.}	247.773	0.55729	newly	409.	564.627	{96., 820.}	67.5977	0.314881	Reff	1.30107	1.56591	{0.883574, 2.34036}	36.1365	ar	6239.	8777.22	{8589., 8894.}	40.6831
34	R0	{1.60764, 10.4729}	{2.1491, 14.6391}	{{2.03831, 13.1693}, {2.22867, 15.6411}}	{33.6804, 39.7807}	inf	658.	1528.39	{1085., 1867.}	132.278	0.355483	newly	455.	754.359	{598., 867.}	66.7205	0.217847	Reff	1.23097	1.26369	{0.849346, 1.7055}	21.9276	ar	6495.	8341.8	{8108., 8575.}	28.4341
35	R0	{1.48527, 5.74485}	{1.9566, 8.42028}	{{1.44762, 4.00571}, {2.33968, 11.4691}}	{32.5438, 60.152}	inf	223.	902.254	{211., 1672.}	312.913	0.59572	newly	365.	794.271	{271., 1178.}	130.722	0.41029	Reff	1.37566	1.536	{0.960055, 2.06859}	25.8091	ar	5811.	7608.24	{5628., 8944.}	35.8645
36	R0	{1.61457, 9.93364}	{1.96553, 11.3384}	{{1.77826, 9.06807}, {2.15363, 13.5067}}	{21.7372, 20.3643}	inf	466.	1145.3	{565., 1741.}	149.413	0.377016	newly	363.	738.783	{442., 912.}	107.718	0.313827	Reff	1.39273	1.32273	{0.887793, 1.78436}	21.4622	ar	6338.	7861.52	{7157., 8392.}	24.0378
37	R0	{1.47831, 7.08139}	{1.95641, 8.76363}	{{1.93419, 8.56054}, {1.93419, 8.56054}}	{32.3413, 23.7558}	inf	307.	975.511	{416., 1466.}	226.405	0.503751	newly	351.	860.319	{458., 1146.}	154.331	0.413335	Reff	1.31939	1.47489	{1.164, 1.75006}	18.0326	ar	5530.	7874.96	{7656., 8582.}	42.4043
38	R0	{1.73445, 9.8858}	{1.50777, 4.77189}	{{1.50982, 4.79544}, {1.50982, 4.79544}}	{13.0691, 51.7299}	inf	459.	534.923	{245., 707.}	36.5008	0.156669	newly	402.	812.346	{455., 1023.}	103.741	0.297034	Reff	1.53534	1.1191	{0.910572, 1.32819}	27.1102	ar	6842.	5823.58	{5611., 6044.}	14.8849
39	R0	{1.66621, 11.4824}	{1.86004, 10.0818}	{{1.77826, 9.06807}, {2.11292, 12.9719}}	{11.6329, 16.9655}	inf	585.	1009.31	{416., 1554.}	86.8594	0.276114	newly	373.	713.2	{344., 920.}	99.2786	0.295024	Reff	1.36629	1.25093	{0.874048, 1.6822}	22.1649	ar	6473.	7544.36	{7105., 8295.}	16.5513
40	R0	{1.60694, 9.95741}	{2.02668, 12.9811}	{{1.88025, 10.9611}, {2.16755, 14.8261}}	{26.1205, 30.3663}	inf	536.	1283.6	{851., 1739.}	142.436	0.380041	newly	419.	721.667	{529., 854.}	75.5851	0.250477	Reff	1.35481	1.30948	{0.871533, 1.67481}	18.638	ar	6625.	8057.84	{7492., 8368.}	21.6278
41	R0	{1.55128, 4.71911}	{1.6546, 4.29593}	{{1.56331, 3.72349}, {1.90341, 5.70177}}	{8.35022, 24.047}	inf	143.	306.975	{20., 634.}	151.031	0.517295	newly	305.	668.725	{32., 1323.}	153.107	0.537163	Reff	1.48892	1.57166	{1.17999, 1.895}	16.2452	ar	6141.	6657.63	{6065., 7995.}	14.8978
42	R0	{1.60063, 6.81118}	{2.26259, 18.845}	{{2.25962, 18.788}, {2.27287, 18.9086}}	{41.3563, 176.678}	inf	336.	718.895	{152., 1553.}	140.753	0.399752	newly	454.	304.684	{73., 592.}	48.3538	0.385427	Reff	1.44265	1.93355	{1.51214, 2.19251}	34.7544	ar	6528.	8554.12	{8463., 8659.}	31.0374
43	R0	{1.56986, 5.44652}	{1.86909, 6.00265}	{{1.83544, 5.78475}, {1.89934, 6.19362}}	{19.1939, 12.5345}	inf	263.	540.127	{82., 1121.}	140.256	0.419907	newly	451.	790.073	{160., 1420.}	107.636	0.361931	Reff	1.45887	1.64604	{1.36612, 1.86743}	16.1254	ar	6312.	7602.69	{7251., 7927.}	20.5894
44	R0	{1.47523, 8.07785}	{2.25406, 14.7424}	{{1.95639, 11.2959}, {2.46976, 17.5057}}	{52.7941, 82.5046}	inf	404.	1011.16	{273., 1665.}	163.97	0.401894	newly	403.	547.266	{163., 907.}	62.3643	0.241636	Reff	1.27976	1.79603	{1.26653, 2.20712}	42.4147	ar	5692.	8547.95	{8173., 8905.}	50.1749
45	R0	{1.6417, 7.69429}	{2.66205, 14.8626}	{{2.35662, 12.2483}, {2.8939, 16.8013}}	{62.1522, 93.1646}	inf	406.	1064.41	{290., 1867.}	172.044	0.397585	newly	444.	623.571	{195., 996.}	62.6255	0.228019	Reff	1.44486	2.17518	{1.75611, 2.56516}	51.148	ar	6735.	9102.46	{8759., 9341.}	35.1516
46	R0	{1.38865, 5.83473}	{1.54221, 5.26743}	{{1.54465, 5.30296}, {1.54465, 5.30296}}	{11.0584, 9.72275}	inf	250.	580.067	{311., 752.}	133.813	0.351904	newly	337.	792.133	{510., 965.}	135.054	0.358728	Reff	1.23465	1.11125	{0.844924, 1.37628}	15.0252	ar	5233.	6094.17	{5836., 6326.}	16.4565
47	R0	{1.45289, 6.87903}	{1.49588, 4.46781}	{{1.49267, 4.47101}, {1.49267, 4.47101}}	{2.95903, 35.0518}	inf	272.	486.375	{282., 659.}	84.1452	0.253404	newly	303.	744.563	{446., 1021.}	145.73	0.368198	Reff	1.28537	1.02916	{0.760665, 1.32325}	21.5888	ar	5379.	5764.91	{5557., 6039.}	7.17431
48	R0	{1.69416, 9.4831}	{2.48422, 19.0349}	{{2.46477, 18.6082}, {2.52006, 19.5955}}	{46.634, 100.724}	inf	453.	722.814	{196., 1367.}	88.562	0.306532	newly	442.	316.8	{98., 593.}	43.8462	0.314308	Reff	1.51407	2.15963	{1.8138, 2.40618}	42.6524	ar	6925.	8903.04	{8833., 8976.}	28.5638
49	R0	{1.47843, 7.16286}	{1.7082, 7.02243}	{{1.54465, 5.30296}, {1.82266, 8.17412}}	{15.5413, 16.2638}	inf	357.	695.935	{114., 1176.}	116.24	0.371273	newly	403.	725.891	{124., 1056.}	100.113	0.345444	Reff	1.2988	1.30792	{1.00155, 1.71726}	17.1739	ar	5449.	6976.52	{5997., 7823.}	28.0331
50	R0	{1.68843, 5.72427}	{1.51531, 2.72346}	{{1.51531, 2.72346}, {1.51531, 2.72346}}	{10.2533, 52.4226}	inf	329.	532.774	{345., 677.}	71.7423	0.261521	newly	521.	1393.45	{1128., 1677.}	172.856	0.444243	Reff	1.53732	1.1176	{0.876152, 1.34635}	27.3019	ar	6800.	5927.94	{5775., 6167.}	12.8245
